# Whether the administration of thyroid hormones may play a role in the treatment of short stature?

**DOI:** 10.1186/1756-6614-8-S1-A10

**Published:** 2015-06-22

**Authors:** Maciej Hilczer

**Affiliations:** 1 Department of Endocrinology and Metabolic Diseases, Polish Mother’s Memorial Research Institute, The Medical University of Lodz, Lodz, Poland

## 

Normal thyroid hormone secretion or optimal substitution of L-thyroxine is necessary for the proper functioning of the hypothalamic-pituitary-IGF-I axis. Over 30 years ago Cacciari et al. [1] indicated a slight risk of inducing an alteration of thyroid function in patients with GH deficiency during hGH therapy and recovery of thyroxine (T4) and trijodthyronine (T3) values to normal limits during follow-up.

The rGH therapy might disclose previously unrecognised thyroid insufficiency rather than induce hypothyroidism [2,3]. The phenomenon of unmasking central hypothyroidism after the beginning of rGH therapy in some children with previous diagnosis of isolated GH-deficiency was described. Changes in TSH secretion during initial phase of the rGH therapy are less evident than fluctuations of FT4 concentration. The possibility of a decrease of TSH in terms of rhGH administration has been explained by an increase of somatostatin, a natural TSH inhibitor. The most frequently quoted mechanism of changes in thyroid hormone levels is GH-mediated increase of peripheral T4 to T3 deiodination. The potential role of IGF-I in stimulating that process has been postulated. Chernausek et al. [4] documented that IGF-I concentrations were diminished in hypothyroid patients. The mechanism of this phenomenon remained unclear. Diminished GH secretion or direct effects of hypothyroidism upon IGF-I production were considered. It is well documented that in patients with congenital hypothyroidism and caused by thyroiditis L-T4 replacement led to physiological increase of IGF-I and IGFBP3 secretion. It was proven that in children with neglected congenital hypothyroidism, even after a long period of hypothyroidism, L-T4 replacement improved the growth rate, leading to a partial recovery of the GH-IGF-I axis [5].

In GH-deficient children from the beginning of rhGH therapy - in euthyroid status - has not been recommended the obligatory L-T4 supplementation due a little evidence for the development of clinically significant hypothyroidism in most of previously euthyroid patients and spontaneous recovery to pre-treatment thyroid function in most patients [6]. Maintaining euthyroid status is important for the best effectiveness of rhGH therapy. The incidence of revealing hypothyroidism should be taken into account while starting rhGH therapy, as hypothyroidism may worsen the poor response to the therapy. It seems that assessment of TSH and FT4 concentration after rhGH therapy onset should be performed earlier and more often - for example every 3 months. It seems important to establish, if possible, the threshold values of pre-rhGH treatment TSH and/or FT4 levels for revealing hypothyroidism during rhGH therapy [7].
